# The incidence and determinants of the meconium-aspiration syndrome among mothers with meconium-stained amniotic fluid after emergency cesarean section: A prospective cross-sectional study in a specialized hospital, south Ethiopia

**DOI:** 10.3389/fped.2023.1149398

**Published:** 2023-03-23

**Authors:** Temesgen Tantu, Dereje Zewdu, Fikretsion Degemu, Tsiyon Yehualeshet

**Affiliations:** ^1^ Obstetrics and Gynecology, Wolkite University College of Medicine and Health Sciences, Wolkite, Ethiopia; ^2^ Anesthesia, Wolkite University College of Medicine and Health Sciences, Wolkite, Ethiopia; ^3^ Pediatrics and Child Health, Wolkite University College of Medicine and Health Sciences, Wolkite, Ethiopia; ^4^ Internal Medicine, College of Health Science and Medicine, Wolkite University, Wolkite, Ethiopia

**Keywords:** MSAF, fetal distress, MAS, prolonged labor, APGAR

## Abstract

**Background:**

Meconium aspiration syndrome is respiratory distress diagnosed in neonates delivered with meconium-stained amniotic fluid that is unexplained by other pathologies. It has severe neonatal respiratory complications and a significant impact on the prevalence of neonatal mortality.

**Objective:**

To identify the incidence and determinants associated with meconium aspiration syndrome among mothers with meconium-stained amniotic fluid after emergency cesarean section in Wolkite University specialized hospitals in Ethiopia from September 1, 2021, to August 30, 2022.

**Method:**

An institution-based cross-sectional study was done prospectively through meticulous chart review and interviews with 275 mothers with meconium-stained amniotic fluid who gave birth with an emergency cesarean section. Data were entered using EpiData 7 and analyzed with SPSS 26. The association between independent variables and the meconium-aspiration syndrome was estimated using an odds ratio with 95% confidence intervals. The statistical significance of the association was declared at a *p*-value of 0.05.

**Result:**

The prevalence of the meconium-aspiration syndrome is 28.7%. The factors associated are: latent phase (AOR: 2.580; 95% CI: 1.126, 5.913), low 1st minute APGAR score (AOR: 2.43; 95% CI: 0.892, 6.625), and thick meconium (AOR: 31.018; 95% CI: 9.982, 96.390). The neonatal death rate associated with meconium aspiration syndrome is 1.8%, and thick meconium contributed to 65% of admissions to the neonatal intensive care unit and all deaths.

**Conclusion:**

The incidence of meconium aspiration syndrome is high, and thick meconium, meconium at early labor, and low APGAR scores all contributed to this. Thick meconium has a substantial effect on neonatal mortality and morbidity. Therefore, an improvement in the quality of obstetric and neonatal care through early intervention in the case of thick meconium and meconium in the early phase of labor is recommended.

## 1. Background

The excretion of fetal intestinal waste known as meconium causes the amniotic fluid to become meconium-stained (MSAF). If the meconium passage is fresh, the amniotic fluid turns greenish; if it is old, it appears yellow. It has been linked to significant neonatal complications (1–4). Meconium aspiration syndrome (MAS) is defined by Cleary and Wiswell as unexplained respiratory distress in neonates with meconium-stained amniotic fluid (5). The incidence of meconium-stained amniotic fluid is 10%–27%, since it varies with gestational age and obstetric complications (1, 2, 6–14). Of the pregnancies associated with MSAF, MAS complicates 5%–20%, depending on the root of delivery (11, 13–16).

The contribution of MAS to neonatal mortality is 3%–5% (6, 8, 17). The pathophysiology of morbidity and mortality is complex and controversial. However, the proposed mechanisms of MAS are airway obstruction, alveolar or parenchymal inflammation, impaired surfactant production and function, infection, and direct toxicity of meconium constituents (18–22). Neonates with MAS are associated with a need for mechanical ventilator support (43.1%), respiratory and metabolic acidosis (30.6%), pulmonary hypertension (11.1%), and hypoxic-ischemic encephalopathy (29.2%) (23).

Several studies have been done to identify the predisposing factors associated with MAS. Risk factors, like operative delivery, thick meconium, a low APGAR score, obstetric complications, fetal heartbeat pattern, post-term pregnancy, and oligohydramnios, are consistently linked with a high likelihood of developing MAS (11–13, 15, 16, 22, 24). On the other hand, contradicting results were observed regarding primiparity, maternal age, and umbilical PH (1, 2, 6, 15).

There have been significant improvements in obstetric care, like intrapartum follow-up, delivery room management, and interventions for fetal distress, in the past decades (15, 18, 22, 23). In addition to obstetric care, there has been a massive advancement in neonatal intensive care units (NICU) and respiratory ventilatory support interventions. Subsequently, this resulted in a progressive decline in the incidence of MAS across the world and an improvement in the neonatal outcome associated with MAS (3, 6). Despite the observed progress in the developed world through evidence-based practice and interventions, neonatal mortality and morbidity secondary to MAS are still problems in the developing world, including Ethiopia (15, 23).

Despite the fact that stakeholders have given neonatal mortality a great deal of attention, there have only been a few studies conducted in Ethiopia that have concentrated solely on the causes of MSAF and its complications. To the best of the authors' knowledge, only one study has been conducted on MAS (25). As a result, there are few statistics on MAS incidence and identifying risk factors in the nation. Moreover, no research has been done on the prevalence and contributing factors of MAS after emergency cesarean sections.

As a result, the primary goal of this study is to determine the prevalence of MAS, and the secondary goal is to identify risk factors for MAS and then to recommend early intervention in pregnancies complicated by the identified risk factors. Thereby, the neonatal complications secondary to MAS can be reduced.

## 2. Materials and methods

### 2.1. Study area, design, and populations

An institutional-based prospective observational study was conducted at Wolkite University Specialized Hospital (WUSH) from September 1, 2021, to August 30, 2022. WUSH is located 160 kilometers southwest of Addis Ababa, Ethiopia's capital. It has been providing service for more than 5 million people in south-central Ethiopia, including the Gurage zone. The monthly delivery rate of the WUSH is 190–210, and of this, 38% gave birth through a cesarean section. And all procedures have been done by experienced obstetricians and gynecologists. All pregnant mothers who had meconium and gave birth through an emergency cesarean section during the study period, as well as those who were eligible, were included in the study.

### 2.2. Sample size determination and sampling technique

The sample size was calculated using a single population proportion by taking the incidence of meconium aspiration syndrome from other studies in Ethiopia [19.9% (11), CI 95%, and margin of error of 5%] to get a total of 275, including the non-response rate. All mothers with meconium-stained amniotic fluid who had an emergency cesarean section and who were eligible were selected by using the convenience sampling method.

### 2.3. Inclusion and exclusion criteria

#### 2.3.1. Inclusion criteria

All mothers who gave birth through emergency cesarean section

#### 2.3.2. Exclusion criteria

Mothers who gave birth by emergency cesarean delivery with one of the following: a fetus with a gross, lethal congenital anomaly that was diagnosed pre-operatively or post-operatively; a negative fetal heartbeat on admission; breech presentation; and mothers who declined or were unable to give a medical history due to any medical disease or obstetric complication.

#### 2.3.3. Operational definitions

**MAS:** diagnosed based on clinical features by a pediatrician (presence of meconium, tachypnea, respiratory grunting, nasal flaring, and chest retractions) and radiographic signs on chest x-rays (patchy infiltrates, hyper expansion) (23).

**Thick meconium**: heavy staining occurs when there is reduced amniotic fluid and a large amount of meconium, making the staining quite thick, with a “pea soup” consistency (15, 16, 26).

**Thin meconium:** amniotic fluid diluted with meconium, with a large to moderate amount of amniotic fluid. Just meconium-stained amniotic fluid (15, 16, 26).

**Latent phase of labor:** the phase of labor starting from the diagnosis of labor up to a four-centimeter cervical dilatation.

**Active phase of labor:** the phase of labor starting from four to ten centimeters of cervical dilatation.

**Second stage of labor:** the period of time between a ten-centimeter cervical dilatation and the delivery of the fetus.

**Prolonged labor:** if labor duration lasts more than 24 h (26).

### 2.4. Data collection instruments

The data collection instruments were derived after an extensive review of the literature. They contain sociodemographic characteristics, obstetric characteristics, perioperative and peripartum characteristics, and early neonatal outcomes. Then, a structured questionnaire was developed and pre-tested at Gunchire Hospital by taking 5% of the sample size for consideration of further modification. Before the commencement of data collection, the data collectors informed the mothers about the purpose and usefulness of the study and then got informed consent. After the above procedure, data collection commenced. The principal investigator recruited three trained midwives to collect data. The training was given to the data collectors, who were supervised daily by the authors. Data was collected through chart review and extensive interviews with the mother. All neonates were followed for the first seven days, and those who were admitted to the NICU were also followed for seven days. Discharged Neonates, who were healthy, were followed by data collectors assigned to specific neonates (midwives) until the seventh day of life with a phone interview with the mother about the neonate, like whether the neonate is breastfeeding, sleeping well, alert, and if there is any problem, the mother was informed to bring it back.

### 2.5. Data processing and analysis

The collected data were entered into Epi Info for data cleaning and error detection before being exported to SPSS 26 for further analysis and association. The result was presented with descriptive frequency distributions, means, graphs, and tables. All variables were analyzed independently through bivariate logistic analysis. The variables with a significant association in bivariate analysis were exported to multivariate analysis for further strength of association, and the significant association was considered if the *p*-value was 0.05 and the 95% CI was included. Model fitness was measured using the Hosmer and Lemeshow goodness of fit measures and the Nagelkerke R square, which were 0.64 and 0.58, respectively. The variance inflation factor (VIF > 10) was used to test for multicollinearity between the explanatory variables.

### 2.6. Clinical set up and practice

**Figure 1:**
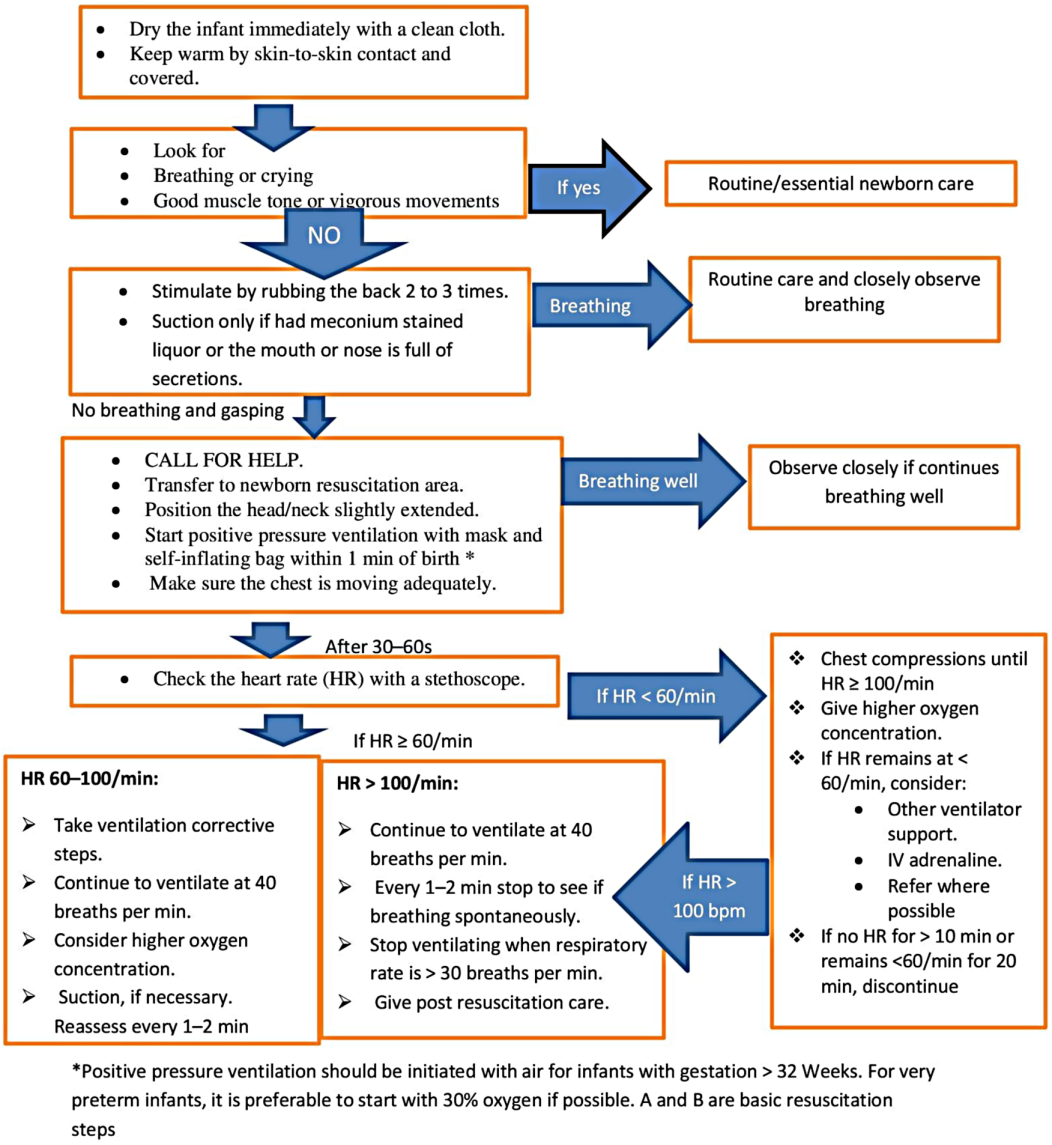
Neonatal resuscitation flow chart.

In this hospital, there are only 2 neonatal nurses, 6 pediatricians, and 5 obstetricians. Obstetricians and midwives handle the majority of neonatal resuscitation in the labor unit, with the help of pediatricians and neonatal nurses as needed. Neonatal resuscitation practices are governed by guidelines created by the Ethiopian federal ministry of health and modified from WHO neonatal resuscitation recommendations. For the vigorous, active, and crying babies with MSAF, the essential newborn care, which is to wrap the baby with a dry towel, continue cord and eye care, provide Vitamin K supplementation, put the baby in skin-to-skin contact with the mother, start exclusive breastfeeding within one hour, and administer vaccinations of BCG, HBV, and polio, will be applied. The Ethiopian guidelines state that the standard neonatal resuscitation guidelines (27) should be used to guide the care of infants with MSAF for any additional interventions, such as endotracheal intubation based on insufficient respiratory effort (gasping, laborious breathing, or poor oxygenation) or heart rate (100 bpm). If the baby with MSAF is not active, not vigorous, and not crying, then immediately check for meconium in the airways and start airway suction under direct vision of the trachea with the laryngoscope and endotracheal intubation if necessary, but gastric lavage is not practiced (Figure 1). Otherwise, routine nasopharyngeal suctioning is not advised for newborns with MSAF who are active, and delayed cord clamping is advised only for babies who are active and do not require resuscitation.

## 3. Results

### 3.1. Sociodemographic characteristics

A total of 275 cases with meconium-stained amniotic fluid were collected. More than 80% of the mothers were in the age range of 20–34 years, and nearly two-thirds were from urban areas (Table 1).

**Table 1:** Sociodemographic characteristics of those mothers who gave birth in Wolkite university specialized hospital in 2022 GC.

Variables (875)	Frequency	Percent
**Family income per month**
<1,000	80	29.1
1,001–4,999	122	44.4
>5,000	73	26.5
**Occupation**
Farmer	32	11.6
Merchant	33	121
Government employee	49	17.8
House wife	153	55.6
Others	8	2.9
**Age**
<20 years	20	7.3
35 years and above	19	6.9
20–34 years	236	85.8
**Place of residence**
Rural	99	36
Urban	176	64
**Current marital status**
Married	271	96.8
Unmarried	4	1.8
Divorced	5	1.4
**Level of education**
Elementary level	74	26.4
High school level	95	33.9
Diploma level	11	3.9
Degree level and above	35	12.5

### 3.2. Obstetrics and health institution-related characteristics

Almost all mothers have the ANC follow-up, and 83.3% have it at the health center or private clinic. Moreover, 39.3% of the pregnant ladies were referred cases, with 59.6% of them from general hospitals. There were nine eclampsia mothers, and most of the cases had a spontaneous onset of labor (Table 2).

**Table 2:** Health institution and obstetric-related factors on those mothers who gave birth at Wolkite university specialized hospital in 2022.

Variables	Frequency	percent
**Gestational age in weeks**
<37 weeks	4	1.5
37–42 weeks	252	91.6
>42 weeks	19	6.9
**Previous history of abortion**
Yes	24	8.7
No	251	91.3
**Previous history of still birth**
Yes	4	1.5
No	271	98.5
**ANC in the current pregnancy**
Yes	271	98.5
No	4	1.5
**Place of ANC**
Health center or private clinic	229	83.3
Primary hospital	42	15.3
Referral Hospital	4	1.4
**History of previous uterine scar**
No	252	91.6
Yes	23	8.4
**Preoperative maternal and/or fetal obstetric complications**
Yes	31	11.3
No	244	88.7
**Preoperative maternal medical complications**
Yes	5	1.8
No	270	98.2
**Admission type by referral**
Not referred	167	60.7
Referred	108	39.3
**Place of referral**
Health center	40	23.4
Government hospital	102	59.6
Private clinic or Hospital	29	17
**Onset of labor**
Spontaneous	267	97.1
Induced	8	2.9
**Eclampsia**
Yes	9	3.3
No	266	96.7
**Preeclampsia**
Yes	13	4.7
No	262	95.3
**Gravidity**
Gravida 1	141	51.3
Gravida 2–4	117	42.5
Gravida 5 and above	17	6.2

### 3.3. Intrapartum and perioperative characteristics

The majority (92.4%) of pregnant mothers had vertex presentation, and 58.5% of surgeries were done during duty time. Accordingly, 52.6% of cases had thick meconium, and the prevalence of meconium aspiration syndrome is 28.3%. And the first and fifth-minute APGAR scores are less than 7 in 25.5% and 17.8% of pregnant ladies, respectively. Furthermore, 16.4% of mothers were in the second stage of labor during the surgery, and 14.9% had labor last more than 24 h. Spinal anesthesia was given to 92.4% of ladies, and the decision to extend the delivery interval was made by more than half of the mothers (52.4%) (Table 3).

**Table 3:** Intrapartum and preoperative characteristics of mothers who gave birth at Wolkite university specialized hospital in 2022 GC.

Variables	Frequency	Percent
**Fetal presentation**
Vertex	254	92.4
Brow	5	1.8
Face	8	2.9
Breech	8	2.9
Shoulder	5	1.8
**Time of operation**
Working hours	114	41.5
Duty hours	161	58.5
**Type of anesthesia**
Spinal anesthesia	254	92.4
General anesthesia	21	7.6
**Maternal blood pressure at decision for operation (SBP/DBP)**
100–139/60–89	201	73.1
140/90 and above	74	26.9
**Maternal blood pressure after anesthesia, but before fetal extraction**
<100/60	4	1.5
100–139/60–89	231	84
140/90 and above	40	14.5
**Type of skin incision**
Pfannenstiel	263	95.6
Midline	12	4.4
**Fetal heat beat patterns**
Brady/tachycardia	16	0.6
Normal FHB	259	99.4
**Surgeon**
Year one resident	13	4.7
Year two resident	179	65.1
Year three resident	73	26.5
Year four resident	5	1.8
Senior	5	1.8
**Sex of neonate**
Male	180	65.5
Female	95	34.5
**Neonatal weight range**
1,000–2,499 grams	20	7.3
2,500–3,999 grams	229	83.2
4,000 grams and above	26	9.5
**1st min APGAR score**
<7	70	25.5
≥7	205	74.5
**5th min APGAR score**
<7	49	17.8
≥7	226	82.2
**Interval b/n decision to delivery**
Greater than 60 min	114	52.4
30–60 min	117	42.5
Less than 30 min	14	5.1
**Stage of labor**
Second stage of labor	45	16.4
Active phase labor	112	40.7
Latent phase of labor	118	42.9
**Duration Labor**
≥24 h	41	14.9
<24 h	234	85.1
**Time taken from referring to managing institution**
Less than 1 h	105	38.3
More than 1 h	169	61.7
**Cephalopelvic disproportion**
Yes	33	12
No	242	88
**Character of MSAF**
Thin	131	47.6
Thick	144	52.6

### 3.4. Perinatal outcomes

The total admission rate to the Neonatal Intensive Care Unit (NICU), the rate of meconium aspiration syndrome, and early neonatal death were 50.2%, 28.7%, and 7.6%, respectively (Table 4).

**Table 4:** Early neonatal outcome of mothers who gave birth at Wolkite university specialized hospital in 2022 GC.

Perinatal outcome	Frequency			Percent
NICU admission	138	50.2
Preterm	13	4.7
Low birth weight	8	2.9
PNA	29	10.5
MAS	79	28.7
Respiratory distress syndrome	9	3.3
Early neonatal death	21 (7.6%)	PNA	16	5.8
		MAS	5	1.8

### 3.5. Factors associated with meconium aspiration syndrome

All variables were fitted independently to a bivariate logistic analysis, and the variables with a *p*-value greater than 0.05 were considered significant. The significant variables from the bivariate analysis were taken to the multivariate analysis to check for further strength of association. According to this study, the variables associated with MAS during bivariate analysis were type of anesthesia given, stage of labor, first minute APGAR score, fifth minute APGAR score, the time between the decisions to deliver, time from reaching the managing institution, duration of labor, the stage of meconium, fetal distress, and cephalopelvic disproportion. Of the above variables, only the following had associations on multivariate analysis: 5th minute APGAR score, stage of meconium, and stage of labor (meconium at latent phase). (Table 5).

**Table 5:** Factors associated with meconium aspiration syndrome on mothers who gave birth to Wolkite university specialized hospital in 2022 GC.

Variables	MAS		COR (95% CI)	AOR (95% CI)	*p*-value
	NO (%)	(YES)			
**Type of anesthesia**
Spinal	187 (73.6)	67 (26.1)	1	1	0.56
General	9 (42.9)	12 (57.1)	3.721 (1.501,9.229)	.309 (.066,1.435)	
**1st minute APGAR score**
<7	37 (52.9)	33 (47.1)	3.083 (1.739,5.466)	2.43 (0.892,6.625)	0.04
≥7	159 (77.6)	46 (22.4)	1	1	
**Stage of labor**
Second	24 (53.3)	21 (46.3)	4.025 (1.883,8.602)	2.27 (0.466,11.038)	0.56
Active phase	80 (67.8)	38 (32.8)	1	1	
Latent phase	92 (82.1)	20 (17.90	2.185 (1.177,4.057)	2.580 (1.126,5.913)	0.025
**Duration of labor**
>24 h	24 (58.5)	17 (41.5)	1.965 (0.990, 3.901)	.968 (0.238,3.941)	0.94
<24 h	172 (73.5)	62 (26.5)	1		
**Time taken from referring institution**
>1 h	109 (64.5)	60 (35.5)	2.661 (1.464,4.835)	1.034 (0.447,2.390)	0.93
<1 h	87 (82.9)	18 (17.1)	1	1	
**Fetal distress**
Brady or tachycardia	8 (50)	8 (50)	2.648 (0.957,7.323)	1.575 (0.667,3.721)	0.3
Normal FHB	188 (72.6)	71 (27.4)	1	1	
**Cephalopelvic disproportion**
Yes	12 (36.4)	21 (63.6)	5.552 (2.575,11.969)	2.143 (0.532,8.626)	0.283
No	184 (77.3)	58 (22.7)	1	1	
**Meconium staging**
Thin	127 (96.9)	4 (3.4)	1	1	0.00
Thick	69 (47.9)	75 (52.1)	34.511 (12.105,98.385)	31.018 (9.982,96.390)	

## 4. Discussion

In this study, the incidence of MAS was 28.7%, which is much higher than other similar studies at different parts of the world (11, 13, 15, 16, 28, 29), but lower than one study done in India and the United States, in which the diagnosis was confirmed with autopsy (10, 30). The NICU admission rate in this study is 50.2%, and of these, 57.2% are diagnosed with MAS. The reason for the higher incidence is the difference in the study populations. The increased rate of cesarean section was reported (13, 15, 28, 29, 31, 32) in pregnancies with meconium-stained amniotic fluid, which can explain the higher rate of MAS in this study because of the already increased baseline meconium-stained amniotic fluid. Moreover, there is evidence from multiple studies showing cesarean section as a risk factor for MAS (6, 18, 23). The study population is pregnant ladies for whom emergency cesarean section was done for obstetric indications.

The thickness of meconium has a statistically significant association with meconium aspiration syndrome. Almost all studies around the world, including this one, link MAS to meconium thickness, though there are a small number of pregnancies with thin meconium that have MAS. Hence, given that thick meconium may potentially restrict the airway, which is supported by other studies, pregnant women with thick meconium are more likely to have MAS than those with thin meconium (11, 17, 24, 29, 33). As a result, thick meconium is more likely to be associated with perinatal mortality (11, 13, 15, 18), and the neonatal deaths rate in this study is 7.6%, with MAS accounting for 1.8% of all deaths. Not surprisingly, all neonatal deaths related to MAS are secondary to thick meconium. Additionally, 65% of neonates delivered with thick meconium are admitted to the NICU, whereas 35% of neonates delivered with thin meconium are admitted to the NICU. This is in line with other studies (2, 10, 11, 13, 14, 18).

Stressful conditions *in utero*, which can be indirectly assessed by a low APGAR score, are implicated in meconium release. Any stressful state in the uterus facilitates the release through relaxation of the anal sphincters and can be associated with fetal hypoxia and subsequent low APGAR scores (4, 34, 35). Furthermore, the fetus with MSAF in the uterus will have a low APGAR score because of the obstruction of the oropharynx by thick meconium and the existing hypoxic insult. As a result, in this study, a low APGAR score is significantly associated with the rate of MAS. Pregnant ladies with low first-minute APGAR scores have higher odds of having MAS than those with an APGAR score greater than 7. Furthermore, a low APGAR score is consistently associated with MAS in the literature (6, 15, 17, 36, 37).

Among obstetric-related factors, the stage of labor is associated with MAS in this study. Having meconium-stained amniotic fluid at an early stage is strongly associated with MAS. This study showed that pregnant women with meconium during the latent phase of labor are more likely to have MAS than those in the active phase. On the other hand, the second stage has no statistically significant association with MAS during multivariate analysis, but it had an association during bivariate analysis. The mechanism for the latent phase to have a strong association may be because of long-duration exposure to meconium-stained amniotic fluid. Moreover, meconium during the early phase of labor may indirectly indicate the long-standing hypoxia that happened before the onset of labor and was diagnosed after the rupture of the membrane during early labor. According to studies, 20%–33% of neonates born with meconium have neurologic and respiratory depression, suggesting chronic exposure to a hypoxic environment (4, 16, 21, 22, 38, 39). Most of the studies associate the stage of labor with the incidence of meconium-stained amniotic fluid (37, 38, 40, 41), but some studies associate labor abnormalities (25) with MAS.

### 4.1. Limitations of the study

The variables like fetal blood acid gas level, umbilical pH, and autopsy for final diagnoses are not included because of a lack of resources to determine them.

### 4.2. Strength of the study

The study tried to include intrapartum, institution-related, and intra-operation-related factors. It is also a prospective study.

### 4.3. Conclusion and recommendation

The incidence of MAS is high in this study. Factors like a low APGAR score, meconium at the latent phase, and thick meconium are identified as factors associated with MAS. Therefore, improving obstetric care like early cesarean sections if there is meconium at the latent phase of labor and neonatal care like advanced neonatal resuscitation and respiratory support is recommended.

## Data Availability

The original contributions presented in the study are included in the article/Supplementary Material, further inquiries can be directed to the corresponding author.
